# Two-Step Preparation of Protein-Decorated Biohybrid Quantum Dot Nanoparticles for Cellular Uptake

**DOI:** 10.3390/pharmaceutics15061651

**Published:** 2023-06-03

**Authors:** Agata Noelia Traverso, David José Fragale, Diego Luis Viale, Octavio Garate, Pablo Torres, Gastón Valverde, Alejandro Berra, Ana Vanesa Torbidoni, Juan Sebastián Yakisich, Mariano Grasselli, Martín Radrizzani

**Affiliations:** 1 Neuro and Molecular Cytogenetics Laboratory, Institute of Emerging Technologies and Applied Sciences (ITECA), National Council for Scientific and Technical Research (CONICET), School of Science and Technology, National University of San Martín, Av. Gral. Paz 5445, San Martín B1650, Argentina; 2 Nanomateriales Funcionales, INTI-Micro y Nanotecnología, Instituto Nacional de Tecnología Industrial, San Martín B1650, Argentina; 3 Science and Technology Institute Cesar Milstein, Fundación Pablo Cassará—National Council for Scientific and Technical Research (CONICET) Saladillo 2452, Ciudad Autónoma de Buenos Aires C1440, Argentina; 4 Translational Laboratory of Immunopathology and Ophthalmology, Department of Pathology, Faculty of Medicine, Universidad de Buenos Aires, Paraguay 2155, Ciudad Autónoma de Buenos Aires C1121, Argentina; 5 Laboratorio de Biología Celular y Molecular, Instituto Argentino de Veterinaria, Ambiente y Salud (IAVAS) Universidad Juan Agustín Maza (UMaza), Mendoza M5519, Argentina; 6 Department of Pharmaceutical Sciences, School of Pharmacy, Hampton University, Hampton, VA 23693, USA; 7 Biotechnological Materials Laboratory (LaMaBio), Department of Science and Technology, National University of Quilmes, GBEyB, Grupo Vinculado IMBICE-CONICET, Roque Sáenz Peña 352, Buenos Aires B1876, Argentina

**Keywords:** nanoparticles, transferrin, endocytosis, drug delivery, albumin

## Abstract

Decoration of nanoparticles with specific molecules such as antibodies, peptides, and proteins that preserve their biological properties is essential for the recognition and internalization of their specific target cells. Inefficient preparation of such decorated nanoparticles leads to nonspecific interactions diverting them from their desired target. We report a simple two-step procedure for the preparation of biohybrid nanoparticles containing a core of hydrophobic quantum dots coated with a multilayer of human serum albumin. These nanoparticles were prepared by ultra-sonication, crosslinked using glutaraldehyde, and decorated with proteins such as human serum albumin or human transferrin in their native conformations. These nanoparticles were homogeneous in size (20–30 nm), retained the fluorescent properties of quantum dots, and did not show a “corona effect” in the presence of serum. The uptake of transferrin-decorated quantum dot nanoparticles was observed in A549 lung cancer and SH-SY5Y neuroblastoma cells but not in non-cancerous 16HB14o- or retinoic acid dopaminergic neurons differentiated SH-SY5Y cells. Furthermore, digitoxin-loaded transferrin-decorated nanoparticles decreased the number of A549 cells without effect on 16HB14o-. Finally, we analyzed the in vivo uptake of these biohybrids by murine retinal cells, demonstrating their capacity to selectively target and deliver into specific cell types with excellent traceability.

## 1. Introduction

One of the most exciting applications of nanotechnology to the biological area involves the use of nanoparticles (NPs) as drug delivery systems. The aim is to take advantage of the benefits of transporting drugs to a target human body region, reducing the systemic toxicity of drugs and increasing their efficacy. Many NPs designed for loading substances have been described, including polysialic acid-based drug delivery systems [1], tumor-specific carrier-free nanodrugs [2], multilamellar vesicles composed of cholesterol and nonionic surfactants, forming a lipid bilayer with an aqueous center (niosomes) [3], metal organic frameworks [4], and hydrophobic ones [5]. Specifically, NPs could modulate the pharmacokinetics, stability, absorption, and exposure of a drug or its combinations, targeting tumors and healthy tissues [6,7].

However, the development of novel nano-constructs able to deliver drugs to specific cells in the human body requires reaching the target, for example, a tumor tissue, without affecting the surrounding normal tissues [8]. The access of NPs to the tumor can also be carried out passively, taking advantage of the increased permeability of the vasculature, changes in the tumor microenvironment (also called EPR effect), or its direct application on the tissue [9]. In addition, specific targeted delivery to cancer cells will decrease the toxicity to non-neoplastic cells [6].

Much effort has been expended to improve NPs’ targeting by decoration with different types of molecules, such as peptides, carbohydrates, receptors, or antibodies that could increase their uptake by tumor cells [10,11]. An example of active transport is that of transferrin (hTf), which crosses the blood-brain barrier [12] and has been used to decorate the surface of human serum albumin (HSA) nanoparticles (NPs) prepared by the desolvation method [13,14] and poly (d,l-lactide-co-glycolide) NPs [15,16].

For many years the effort of many researchers into NP targeting has been hampered by the ‘corona effect’ masking the original chemical or biological functionalities of the recognition molecule [17]. Recently, we described novel protein/gold biohybrid NPs containing a chemically stable multilayer of albumin coating [18,19]. This procedure was based on radiation-induced protein crosslinking [20] and further decoration with specific peptides for cell targeting [21]. The nanoconstruct was able to improve target-cell recognition and reduce nonspecific cell interaction in in vitro cell cultures [21]. Radiation-induced crosslinking introduces protein modifications [22] which can induce immune response [23]. To overcome this drawback, a surface decoration with native HSA was proposed [21,24] which shows an enhanced cell uptake property [21]. However, the preparation procedure becomes a multistep and low-yield process.

The aim of the present work was to simplify the preparation of biohybrid NPs and also simplify their tracking. Cadmium selenide core with zinc sulfide shell quantum dots (QDs) were selected for their optical properties which allow tracking single NPs on living cell culture using a fluorescence microscope. Therefore, we describe a two-step preparation of a decorated biohybrid NPs (QDs + albumin multilayer + albumin or transferrin monolayer shell) using a combination of a two-phase system and ultrasound emulsion. These surface-decorated biohybrid NPs could deliver drugs and target human cell lines in a measurable and traceable way. Efficient in vivo cellular uptake was demonstrated in a murine retina.

## 2. Materials and Methods

### 2.1. Materials

QDs solutions at 5 mg/mL (1.0 nmol/mg) with emission wavelengths at 525 nm and 630 nm, corresponding to green (Cat #: QD-525-A-5MG) and red (CAT #: QD-630-A-5MG) fluorescence emissions, respectively, were purchased from Cytodiagnostics Inc. (Burlington, ON, Canada). Human serum albumin (HSA, 200 mg/mL) was kindly donated from the Laboratorio de Hemoderivados, Universidad Nacional de Córdoba (Córdoba, Argentina). Human transferrin (hTf) and alkaline phosphatase (AP) were purchased from Sigma-Aldrich (St. Louis, MO, USA).

### 2.2. Methods

#### 2.2.1. Preparation of Water Soluble Biohybrid QDs

Water soluble QDs were obtained by mixing chloroform: methanol (4:1) 1 mL + QDs (5 mg/mL) 20 μL followed by gently adding to the aqueous phase PBS 350 μL + HSA (10 mg/mL) 350 μL + glutaraldehyde (5%) 1.5 μL + H_2_O 298.5 μL. The mixture was sonicated using an ultrasonicator SONICS Vibra Cell VCX50 (Oklahoma City, OK, USA) (6 mm probe, amplitude of 40%) until the solution reached 60 °C. The suspension was immediately centrifuged at 10,000× *g* for a few seconds and the upper water phase containing water-soluble glutaraldehyde-activated biohybrid QDs (gluta-QDs) was recovered.

#### 2.2.2. Decoration of Biohybrid HSA-QD NPs

The gluta-QDs (QDs coated with a protein multilayer shell) were decorated by incubation with hTfs (5 mg/mL) at room temperature overnight (ON). The remaining reactive sites were blocked Tris-Glycine 1 M, pH 8.8. These decorated gluta-QDs with hTfs (hTfs-QDs) were recovered by centrifugation at 13,000× *g* for 10 min. The supernatant was discarded and hTfs-QDs were resuspended in 500 μL of Milli-Q H_2_O. Visualization under Ultraviolet Light C (350 nm) was used as a quality control to monitor the preservation of the fluorescence emitted by the QDs in the NPs after each preparation.

Using the same procedure, gluta-QDs were decorated with HSA (5 mg/mL) or alkaline phosphatase (AP, 1 mg/mL) to obtain HSA- and AP-QDs respectively.

### 2.3. Characterization of Decorated NPs

#### 2.3.1. Tyndall Effect

The decorated NPs were diluted (1:1–1:10) in PBS and analyzed by the incidence of a red-wavelength laser to show the Tyndall effect due to light scattering by the presence of NPs in the solution.

#### 2.3.2. Dynamic Light Scattering (DLS)

Hydrodynamic diameters of decorated NPs were measured on 100 μL diluted samples by Dynamic Light Scattering (DLS) in the Wyatt Dyna-Pro reader (Dernbach, Germany) and analyzed with Dynamics Software v7.8. Between 500,000 and 2,000,000 events (10 acquisitions, 5 s/each) were studied at 25 °C.

#### 2.3.3. QDs Fluorescence

A sample of 1.5 μL of decorated NPs suspension was placed on a slide and observed in an Olympus X71 (Tokyo, Japan) fluorescence microscope with light and a UV filter. The QDs (red) were excited with white light, UV light, and blue light, and the absorption spectra were measured using a Nanodrop 3300 spectrofluorometer.

#### 2.3.4. Electrophoresis

Water-soluble NPs were analyzed by electrophoresis on low melting Agarose gel 0.8%, using 0.5× TBE buffer. Fluorescamine (FSC) 1% was added to the samples to detect the green fluorescence revealing the presence of reactive amino moieties in proteins.

#### 2.3.5. Transmission Electron Microscopy (TEM)

Diluted suspensions of decorated NPs were fixed with glutaraldehyde (0.7% final concentration) for 30 min. Fixed NPs were mounted in grids, stained with Uranyl acetate (2% final concentration), and analyzed in a Zeiss EM 109-T Transmission Electron Microscope (TEM) (Oberkochen, Germany) equipped with a Gatan ES1000W (Warrendale, PA, USA) digital camera at the Instituto de Biología Celular y Neurociencia, LANAIS-MIE (UBA-CONICET).

#### 2.3.6. Confocal Laser Microscopy

Cell cultures were observed using a Carl Zeiss LSM 800 confocal laser scanning microscope (Zeiss, Oberkochen, Germany). For each sample, three image stacks were taken with a z-step size of 1 μm. Unstained and single-stained slices for each dye (red or green) were used to monitor and subtract all respective background signals. The Zeiss ZEN Microscope Software version 3.0 and COMSTAT 2.1 (www.comstat.dk, accessed on 26 April 2023) was used for the generation of orthogonal and 3D images [25].

#### 2.3.7. Quantification of Decorated NPs in Solution

Serial dilutions (1/10, 50 μL each) of QD solution made in solvent (Cl4C), from commercial QDs (20 nM), were used as the standard to create a fluorescence calibration curve and measure the concentration of decorated NPs by interpolation. The fluorescence of each sample was measured in triplicates using the fluorescence capability of a real-time PCR (Step One, Applied Biosystems). The results obtained were corroborated by counting under a microscope at 1000× using 2 μL samples of each dilution, which were left to dry on a coverslip and subsequently digitized. The quantification was performed using ImageJ software and by averaging 10 different areas taken at random. The regression fit and its dispersion were also calculated using least squares fitting.

#### 2.3.8. Quantification of Decorated NPs Uptake in Cell Culture

Ten homogeneous fields with cells per glass were counted. Each cell containing HSA-QDs (green) and hTf-QDs (red) was distinguished. The results were statistically analyzed with the *t*-test, confidence intervals for ratios, and a paired samples test to validate the results. Cellular uptake of the NPs was analyzed using R software and the ‘boxplot’ tool.

#### 2.3.9. Preparation of Digitoxin-Loaded hTf-QDs

hTf-QDs NPs (2 nM in Phosphate buffer, 0.1 M, pH = 7) were incubated for 72 h in ethanol and a digitoxin-saturated solution (1.35%) in a ratio of 3:2. The mixture was centrifuged and washed twice with phosphate buffer 500 μL. The hTf-QDs-Dix were resuspended in serum-free culture media as a stock solution (200 pM) and stored at 4 °C until use. Control hTf-QDs NPs were prepared using the same procedure with ethanol but without digitoxin.

#### 2.3.10. Cell Lines

A-549 alveolar cancer cells [26] originally obtained from a culture of type II lung carcinomatous tissue explants were purchased from the American Type Culture Collection (ATCC) collection (A549 CRM-CCL-185). Cells were seeded and propagated in complete medium: DMEM high glucose, supplemented with fetal bovine serum (FBS) 5%, L-glutamine 2 to 4 mM, penicillin 100 U/mL, and streptomycin 10 µg/mL. All cells were cultivated with CO_2_ 5% at 37 °C.

The noncancerous transformed non-neoplastic bronchial epithelial cell line 16HBE14o- was purchased from Sigma-Aldrich [27] and used as a control. This cell line derives from the human bronchial epithelium and has been transformed with the T-SV40 antigen for its immortalization. 16HBE14o- cells were grown and propagated in Eagle’s minimal essential medium (MEM) supplemented with FBS 10%, L-glutamine 2 to 4 mM final, penicillin 100 U/mL, and streptomycin 10 µg/mL.

Neuroblastoma SH-SY5Y cells (obtained from ATCC, CRL-2266™) were routinely cultured (175 mL flasks) in minimum essential medium (MEM) supplemented with heat-inactivated FBS 10%, L-glutamine 2 mM, penicillin 50 U/mL, and streptomycin 50 μg/mL in a humidified CO_2_ 5% incubator at 37 °C. For experiments, 80,000 cells in a 70 μL volume were allowed to adhere in the center of the coverslip for two hours. Then, the volume was adjusted to 1 mL. SH-SY5Y cells were differentiated in neurons by retinoic acid (RA, Sigma-Aldrich) treatment as described by [28]. Briefly, adherent SH-SY5Y cells were incubated with medium containing SFB 1% and retinoic acid (RA) 10 μM (differentiation medium) for seven days. The culture media were replaced twice a week.

The retinal pigment epithelium (RPE) cell line ARPE-19 was purchased from the American Type Culture Collection (ATCC) collection (ARPE-19, CRL-2302). ARPE-19 cells were cultivated in DMEM:F12 medium with fetal bovine serum to a final concentration of 10%, sodium bicarbonate 56 mM final concentration, and L-glutamine 2 mM. All cells were grown with CO_2_ 5% at 37 °C.

Incubation of decorated NPs in cell cultures was performed in 24-well plates in 500 μL of the appropriate media (8 pM final concentrations, 3 h incubation time or 20 pM, 72 h incubation time). After incubation, cells were washed once with pre-warmed (37 °C) culture media followed by two washes with PBS (37 °C). Cells were fixed for 20 min with formaldehyde 4%, and washed twice with H_2_O 500 μL. For fluorescence microscopic visualization, the slides were covered with DAPI-containing antifade mounting solution (ProLong^®^ Gold antifade reagent, Invitrogen).

#### 2.3.11. In Vivo Analysis of NPs Distributions

BALB/c mice (7 to 8 weeks old) received a subconjunctival injection of a mix of HSA-QDs (green) and hTf-QDs (red) in 10 μL (100 pM each) using a 32-gauge syringe (Hamilton, Reno, NV, USA). Control mice were injected with 10 µL of PBS. Both eyes of the animal received identical treatment. Animal use and care procedures were approved by the Institutional Animal Care and Use Committee of the University of Buenos Aires. All experimental procedures were in agreement with the ARVO Statement for the Use of Animals in Ophthalmic and Vision Research.

Tissue processing: Animals (three per group, total 12) were sacrificed at 24 h post injection, and the eyes were harvested and fixed with paraformaldehyde 4% in 0.1 M phosphate buffer, pH 7.4, for 24 h. In another group, free, extracellular NPs were washed by perfusing with PBS 1x prior to fixation with paraformaldehyde and the eyes were isolated [29]. Retinal sections (16 μm thick) were obtained using a cryostat, stained with DAPI, and observed with a confocal or fluorescent microscope [29].

## 3. Results

### 3.1. Preparation and Characterization of Decorated NPs

**Figure 1:**
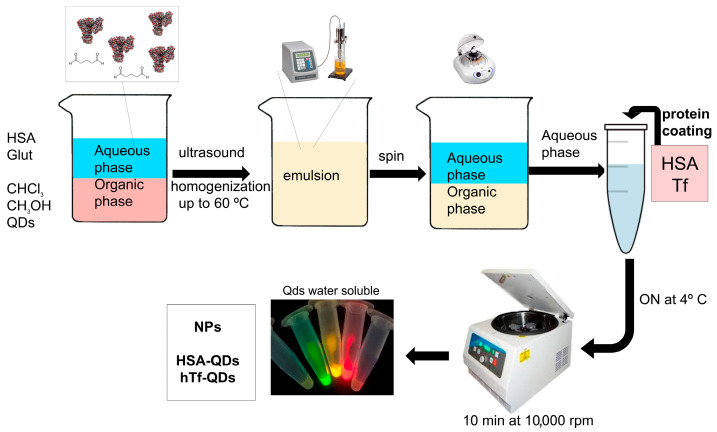
Preparation of decorated NPs. The first step is the preparation of a biphasic system, followed by the application of ultrasound, and phase separation by centrifugation. The process takes 20 min and provides NPs in an aqueous phase, with their surface activated. In the second step, NPs were decorated by the addition of a protein or peptide of interest to the solution and incubating ON at 4 °C followed by centrifugation at 10,000 rpm. The picture shows the resuspended, water-soluble decorated HSA-QDs illuminated with UV light, with turquoise (450 nm), green (525 nm), yellow (570 nm), and hTf-QDs with red (630 nm), and infrared (780 nm) emissions.

The hTf- and HSA-decorated NPs (hTf-QDs and HSA-QDs, respectively) were prepared by following the scheme shown in Figure 1 that summarizes the coating procedure described in Section 2. When the same procedure was performed at 4 °C during the sonication, almost all NPs remained in the organic phase, reaching lower yield in the aqueous phase. The best yield was obtained when phosphate buffer pH 7.2 with ionic strength between 62.5 and 125 mM of NaCl was used.

**Figure 2:**
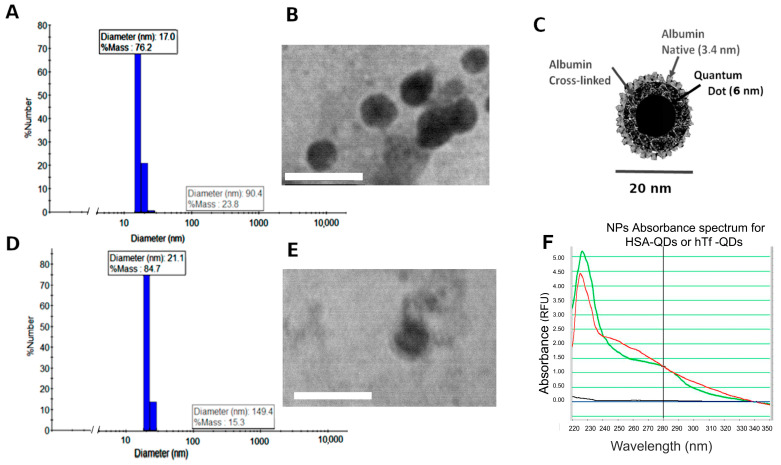
Characterization of decorated NPs. The dynamic light scattering (DLS) plots show the relative mass and media diameter of HSA-QDs (**A**) and hTf-QDs (**D**). The insets are TEM images (100,000×) of HSA-QDs (**B**) and hTf-QDs (**E**), respectively, after uranyl acetate staining. The scheme in (**C**) represents the structure of the NPs obtained with this procedure. (**F**) Absorbance (RFU = Relative Fluorescence Units) spectra of HSA-QDs (green) and hTf-QDs (red) obtained using a Nanodrop 1000 spectrophotometer.

The hydrodynamic radius of the decorated NPs was analyzed by DLS. The mean diameter of HSA-QDs and hTf-QDs was 17 nm and 21 nm, respectively (Figure 2A,D). TEM images agree with the mean diameters recorded by DLS technique (Figure 2B,E). It was also observed that hTf-QDs showed higher contrast than HSA-QDs. In the HSA-coated NPs, higher intensity can be observed in the core, corresponding to a QD (Figure 2B,C). Another difference was the aggregation of the hTf-QDs in the sample when it was dehydrated for TEM. The aggregation is likely due to the formation of hTf dimers in the solution as has been described previously [30].

Using geometrical relationships, it was possible to estimate the composition of the decorated NPs. Taking the diameter of HSA-QDs or hTf-QDs to be approximately 20 nm, and assuming they have a spherical shape, each NP will have a volume of approximately 4000 nm^3^. Subtracting the volume of a QD in the core (around 200 nm^3^), 3800 nm^3^ will be filled by proteins: considering the albumin volume of 120 nm^3^ [23], approximately 32 HSA molecules per NP. According to experimental data from the literature [23,25], the first inner layer will be four HSA molecules to cover the QD surface. Surrounded by the inner layer, at least two layers of denatured HSA complete the NP core, which is decorated with native proteins, HSA, or hTf (Figure 2C). Higher-size NPs could be assigned to aggregates of these 20 nm NPs.

**Figure 3:**
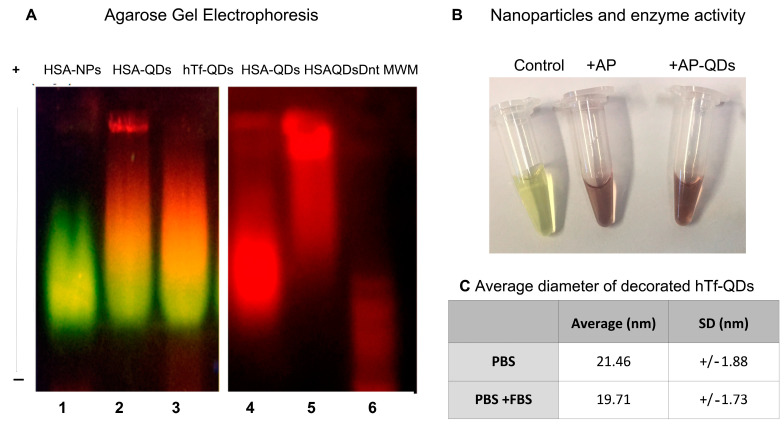
The external monolayer shell of decorated NPs preserves protein function. (**A**) Left panel: UV-illuminated agarose gel electrophoresis of HSA-QDs (lane 2) and hTf-QDs (Lane 3). HSA-NPs without QDs were used as control (lane 1). Right panel: UV-illuminated agarose gel electrophoresis of HSA-QDs (lane 4) and heat denatured (5 min at 95 °C) ones (HSAQDsDnt, lane 5). Free amines were labeled with FSC to visualize HSA (green), hTf (green). QDs are observed by their own emission (red). Lane 6 shows a DNA ladder (100–3000 bps) visualized by the presence of ethidium bromide (red). (**B**). Activity (brown color) of AP-QDs (Tube 3, AP-QDs). Reactions without enzyme (yellow color, tube 1) or with AP (1 mg/mL, tube 2) were used as negative and positive control, respectively. (**C**) Average diameter of hTf-QDs in PBS alone or PBS + FBS 50% demonstrating lack of corona effect.

The decorated QDs were further characterized by agarose gel electrophoresis. As in the case of macromolecules, water-soluble QDs can run in electrophoresis gels, where proteins are detected by the green (525 nm) and the QDs by a red (630 nm) fluorescent signal. The samples in the left gel (Figure 3) were prestained with FSC. As expected, fully assembled decorated QDs showed a yellow color due to the colocalization of both green and red colors (Figure 3A left gel, lanes 2 and 3). As a control, the first lane corresponds to HSA-NPs prepared by omitting the addition of QDs in the procedure. HSA-QDs and hTf-QDs show electrophoretic mobility comparable to HSA-NPs. Because HSA denaturation occurs at a temperature >56 °C [31], the external HSA layer of decorated NPs was heat-denatured at 95 °C for 5 min (Figure 3A right gel, HSAQDsDnt, lane 5). The delayed mobility observed only in lane 5 due to the thermal treatment indicates that our preparation method preserves the native structure of albumin present in the external layer. The latter is relevant because the native conformation of a protein is required for its biological activity. The molecular weight marker (100 bp ladder, range 100–3000 bps) prestained with ethidium bromide shows that HSA-QDs have a mobility higher than 3000 bps. Finally, we further analyzed the preservation of the native structure of a protein by preparing gluta-QDs decorated with alkaline phosphatase (AP-QDs) followed by the detection of its enzymatic activity. Figure 3B shows that the enzyme is active on the NP’s surface. In addition, absorbance spectra of the HSA-QDs and hTf-QDs were plotted after the NPs were washed by centrifugation/dilution steps. Finally, hTf-QDs incubated for 30 min in PBS +/− FSB showed a similar size indicating a lack of the corona effect (table in Figure 3C).

### 3.2. Fluorescence Stability of Decorated NPs

**Figure 4:**
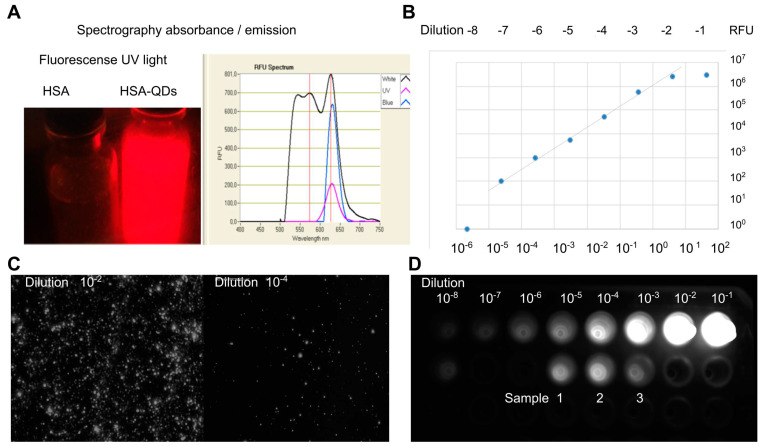
Biohybrid decorated NPs preserve the fluorescent properties of QDs (**A**) and can be quantitatively measured by fluorimeter (**B**), fluorescence microscopy (**C**), or image analysis (**D**). (**A**) Left panel: Fluorescence (630 nm emission) of 20-mL flasks containing HSA-NPs (left) and HSA-QDs (right) illuminated with UV light at 350 nm. Right panel: absorbance spectra plot of HSA-QDs measured using a Nanodrop 3300 fluorimeter. (**B**) Fluorescent quantification (Relative Fluorescent Unit, RFU) of HSA-QDs excited with white light, UV (280–350 nm) light, and blue (490 nm) light measured on Nanodrop 3300. (**C**) Representative microscope fluorescent images of diluted solutions (10^−2^ and 10^−4^) dried onto coverslips of HSA-QDs. (**D**) Quantification of HSA-QDs samples by image analysis. A serial dilution (10^−1^–10^−8^) of QDs was used as standard. UV excited fluorescence of QDs was captured by a LAS 500 digital analyzer and quantified by extrapolation using Image-J software.

Coating and ultrasound treatments could affect the fluorescence-emission properties of QDs. Therefore, to determine if the coating procedure caused any interference in the QD’s fluorescence its light emission spectra were recorded. The decorated NPs’ fluorescence was measured by spectrofluorometer, compared with the QDs (in solvent), and brought to the same concentration using the fluorescence intensity. The QDs’ dilutions showed similar fluorescence intensity without significant differences (Figure 4A,B). In addition, QDs were quantified by serial dilutions under the microscope. A constant volume of each QD’s dilution was placed on a coverslip and dried. Figure 4C shows a picture of HSA-QDs obtained with an inverted fluorescence microscope (1000×). The accompanying video (Supplemental Material Video S1) shows that decorated NPs (1600×) have similar random motion properties (Brownian Movement) in solution.

The concentrations of QDs were obtained by multiplying the dilution factor by the number of counted QDs in the total area. Both measurements predict the same concentrations of QDs. The results obtained with the samples of the QDs decorated with HSA and hTf were interpolated in the scale prepared from QDs in solvent. The samples were compared using fluorescence images obtained in the LAS 500 equipment and quantified with ImageJ (Figure 4D). The samples 1, 2 and 3, yielded concentrations of 40, 50, and 100 nM in each sample, respectively. The data indicate that decorated NPs preserve the fluorescent properties of QDs.

### 3.3. Cellular Uptake of HSA-QDs and hTf-QDs

The corona effect that results from the non-specific binding of serum protein to the surface of NPs has been described as a barrier that prevents the effective uptake and delivery of NPs and their cargo [32]. We previously demonstrated the HSA multilayer gold NPs do not show a corona effect. In addition, these NPs have been used in vitro in uptake experiments in cell culture with media containing SFB 10% [21]. To address this issue, we performed uptake experiments in A549 and SH-SY5Y cell lines.

SH-SY5Y neuroblastoma cells can be grown in their native form or as differentiated dopaminergic neurons when treated with Retinoic acid for 10 days. Native neuroblastoma and differentiated dopaminergic neuron cells express different levels of HSA and hTf receptors in the cell membrane. Therefore, this system offers an excellent model to study the differential uptake of HSA-QDs and hTf-QDs [33,34].

**Figure 5:**
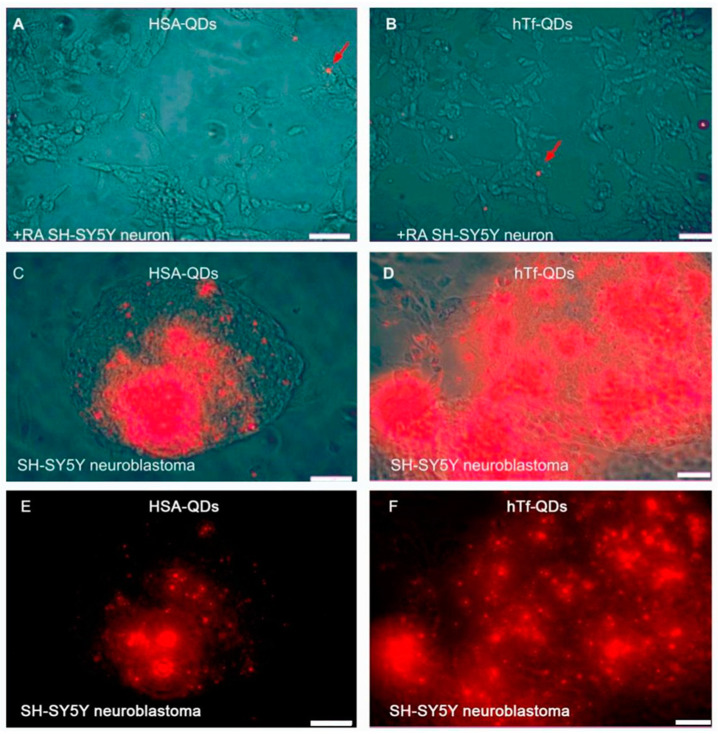
Early and selective uptake of HSA-QDs and hTf-QDs in neuroblastoma cells. Human SH-SY5Y neuroblastoma line differentiated into dopaminergic neurons by retinoic acid (+RA) does not internalize HSA-QDs (**A**) or hTf-QDs (**B**) after 72 h incubation. In contrast, SH-SY5Y neuroblastoma cells show strong fluorescent signals after only 3 h of incubation with HSA-QDs (**C**) or hTf-QD (**D**). (**E**,**F**): UV Fluorescence signals of (**C**,**D**), without contrast light. Red arrows indicate nonspecific NP interactions. Bars = 50 µm.

Figure 5A,B show an absence of uptake to differentiated cells (dopaminergic neurons) after 72 h incubation for both NPs at 20 pM final concentrations in complete media. Meanwhile, at only 3 h incubation it can visualize an uptake of the decorated NPs to the native neuroblastoma cells. In addition, higher hTf-QDs income is recorded than those decorated with HSA (Figure 5C,D).

**Figure 6:**
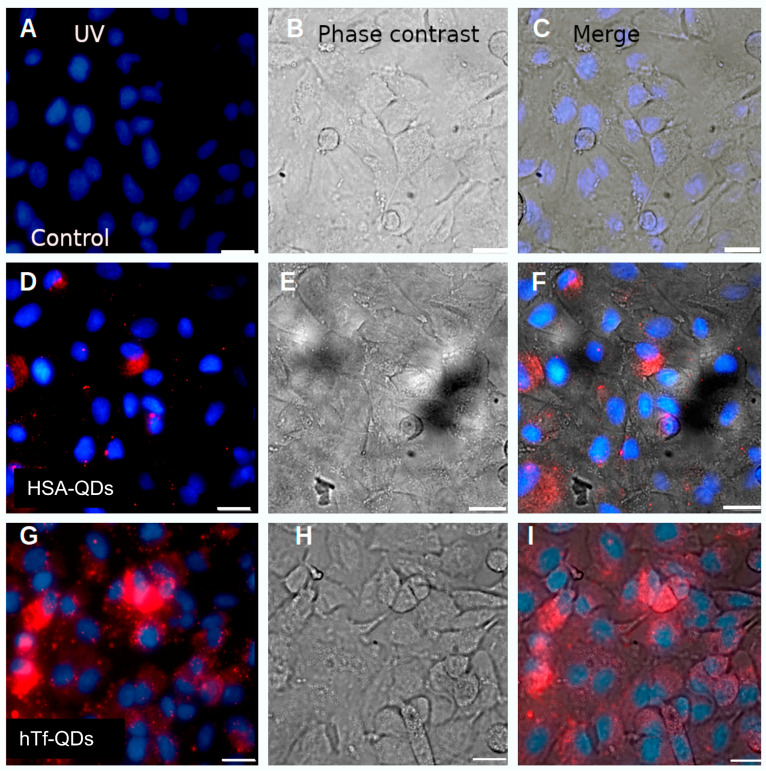
A549 lung cancer cells internalize HSA-QDs and hTf-QDs. Fluorescence microscopy representative images of A549 cells. Fluorescence emitted with ultraviolet light (UV) (First column; (**A**,**D**,**G**)), phase contrast (second column; (**B**,**E**,**H**)) and merge (third column; (**C**,**F**,**I**)). The first row shows cells incubated without NPs. The second and third rows were incubated with HSA-QDs and hTf-QDs, respectively. Scale bars = 20 μm.

We also studied the uptake of decorated NPs in A549 lung adenocarcinoma cells. This cell line can take up hTf in a concentration-dependent and competitive fashion [35]. It is expected this protein could be driving the NP uptake to this cell line. Figure 6 shows that A549 cells were able to incorporate HSA-QDs and hTf-QDs (final concentration of 8 pM) in complete media (containing FBS 5%). As expected, a larger quantity of those decorated with hTf was observed (Figure 6G–I) in comparison to HSA-decorated ones (Figure 6D–F).

**Figure 7:**
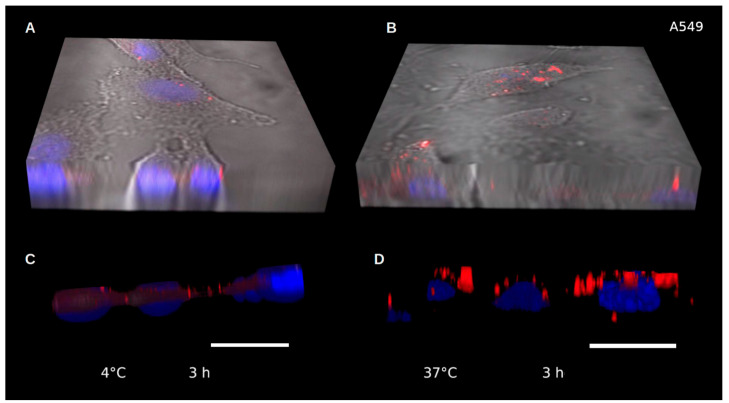
Energy-dependent internalization of HSA-QDs. Reconstruction of 3D images captured by confocal microscopy after incubation for 3 h with HSA-QDs at 4 °C (**A**,**C**) or 37 °C (**B**,**D**), respectively. HSA-QDs and DAPI-stained nuclei are shown as red and blue, respectively. Scale bars = 20 μm.

To demonstrate the subcellular localization and its dependency on an active energy-dependent process we evaluated the cellular uptake of decorated NPs in A549 cells at 37 °C and 4 °C using confocal microscopy. Figure 7 shows 3D confocal image reconstruction of A549 cells incubated with HSA-QDs at 37 °C and 4 °C for 3 h. The outer membrane was labeled with Fluorescein isothiocyanate (FITC) as faintly observed. At 4 °C few small-size NPs remain on the cell surface, but no decorated NPs were observed intracellularly (Figure 7A,C). At 37 °C, HSA-QDs were found in the cytoplasm, below the cell membrane, and concentrated around the cell nucleus (Figure 7B,D).

### 3.4. Quantification of Cellular Uptake

**Figure 8:**
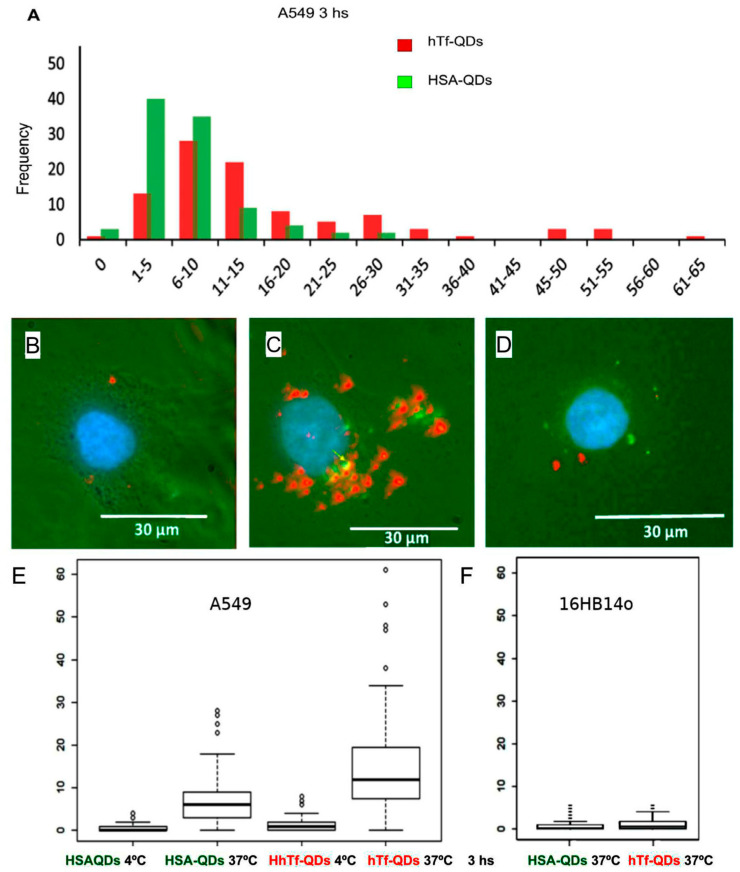
Temperature-dependent incorporation of hTf-QDs is higher than HSA-QDs in A549 cells. Cells were co-incubated with hTf-QDs (red) and HSA-QDs (green) for 3 h. (**A**) Representative plot showing the frequency of the number of NPs per A549 cell (quantification of three independent experiments are shown in panels (**E**,**F**)). (**B**–**D**) Representative fluorescence microscopy images using UV and transmission light simultaneously of A549 cells at 4 °C (**B**), 37 °C (**C**), and 16HB14o- cells at 37 °C (**D**). The co-localization of two HSA-QDs and hTf-QDs is visualized in yellow (arrow). DAPI-stained nuclei are shown in blue. (**E**) Box plot of HSA-QDs and hTf-QDs per A549 cell incubated at 4 °C or 37 °C during 3 h. The box comprises the 2nd and 3rd quartile of the data and the horizontal thick line defines the median. Outliers (>1.5× interquartile range are marked by diamonds). (**F**) Box plot of the average number of HSA-QDs and hTf-QD per 16HB14o- cell incubated at 37 °C for 3 h.

In order to obtain quantitative data of the cellular uptake, HSA-QDs and hTf-QDs (green and red, respectively) were counted in the cytoplasm region of each cell after 3 h incubation time. Figure 8A shows the histogram plot for HSA-QDs and hTf-QDs. The cellular uptake of hTf-QDs (red) was higher than HSA-QDs (green). Fluorescence microscopy also shows that there are minimal co-localization events of NPs within cells and when it is found, it is close to the cell nucleus. A similar experiment was performed on 16HB14o- cells, a non-cancerous human line of immortalized tracheolae (Figure 8D), where a very low number of NPs were found. For comparison, the differential uptake of HSA-QDs and hTf-QDs in A549 and 16HB14o- cells at 4 °C and 37 °C is shown in Figure 8E,F. In A549 cells at 37 °C, an average of 8.2 hTf-QDs/cell more than HSA-QDs was found. In contrast the average uptake at 4 °C was 0.46/cell. In the normal 16HBE14o- cells, no significant difference was observed between hTf-QDs and HSA-QDs at 37 °C.

### 3.5. Ability of hTf-QDs to Deliver Cargo into Cells

**Figure 9:**
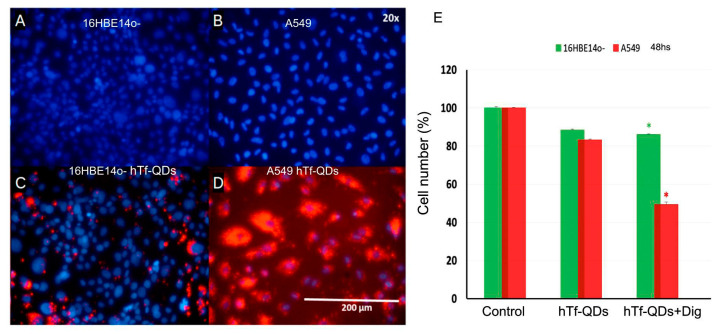
Digitoxin-loaded hTf-QDs reduced A549 cell number. Left panel: Representative fluorescence microscopy images (20×) of 16HB14o- and A549 cells incubated for 48 h without (Control, (**A**,**B**) panels) or with hTf-QDs ((**C**,**D**) panels). hTf-QDs are visualized in red and DAPI-stained nuclei are shown in blue. (**E**) Effect of hTf-QDs loaded with digitoxin (hTf-QDs-Dig) on 16HB14o- (green) and A549 (red) cells treated for 48 h. As a control, cells were incubated without hTf-QDs (Control) or in the presence of hTf-QDs without digitoxin (hTf-QDs). * indicates significative difference between treatments.

Our previous data demonstrated that hTf-QDs were superior to HSA-QDs in terms of cellular uptake. For this reason, we evaluated the ability of hTf-QDs to deliver digitoxin. Digitoxin was selected as a model hydrophobic molecule to be selectively transported to cells. Previous studies have shown that digitoxin inhibits the growth and induces apoptosis of cancer cells at nanomolar concentrations [36,37]; in particular, digitoxin inhibits the Src pathway in the A549 cell line [38]. This molecule has very low solubility in water, but it is soluble in ethanol 100%. Therefore, digitoxin was loaded into hTf-QDs by incubation in an aqueous/ethanol solution of digitoxin and purified. A549 and 16HB14o- cells were incubated with hTf-QDs loaded with digitoxin (hTf-QDs-Dig) for 48 h. After 48 h incubation (Figure 9) a large accumulation of hTf-QDs-Dig was observed in A549 cells with a concomitant significant reduction (>50%) in cell number. In contrast, accumulation of hTf-QDs-Dig in 16HB14o- cells was negligible and non-significant effect on cell numbers was observed (Figure 9E). hTf-QDs without digitoxin were used as control.

### 3.6. In Vitro and In Vivo of NPs Distributions in Ocular Retina

**Figure 10:**
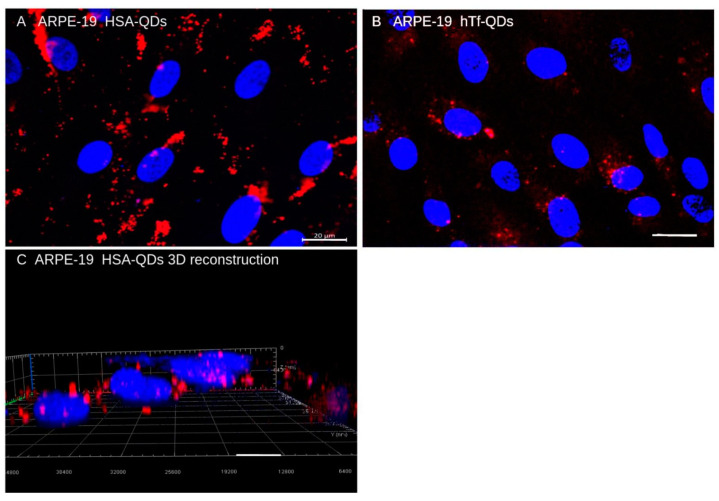
Uptake of HSA-QDs (Panel (**A**)) and hTf-Qs (panel (**B**)) by ARPE-19 cells. The 3D reconstruction demonstrates HSA-QDs aggregates in the cytoplasm (Panel (**C**)). All images (merge) were taken with red and blue (DAPI) filters. Scale bar = 20 μm.

We first analyzed the distribution of NPs in ARPE-19 cells. Figure 10 shows that ARPE-19 cells incorporate HSA-QDs (Panel A) and, to a lesser extent, hTf-QDs (Panel B). The 3D reconstruction (Panel C) demonstrates that HSA-QDs form aggregates in the cytoplasm.

**Figure 11:**
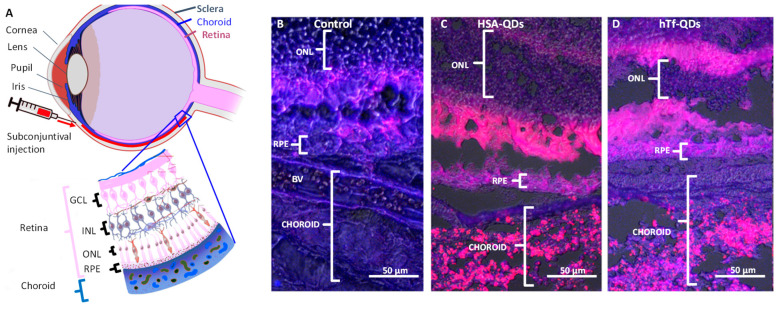
In vivo circulation of decorated HSA-QDs and hTf-Qs by RPE cells via subconjunctival injection. (**A**). Schematic of the eye anatomy and retina and subconjunctival injection of NPs. GCL = Ganglion Cell Layer, INL = Inner Nuclear Layer, ONL = Outer Nuclear Layer, RPE = retinal pigment epithelium (**B**). Control tissue of eye inoculated via subconjunctival injection with PBS showing spontaneous autofluorescence (BV = Blood vessel). (**C**,**D**) Tissue inoculated via subconjunctival injection with HSA-QDs and hTf-QDs, respectively. All images were taken with a red filter and phase contrast (merge). Scale bar = 50 μm.

We next inoculated via subconjunctival injection Balb/c mice with normal retinas with 10 μL of NPs suspension using a Hamilton syringe and a 32G needle. After 24 h, the animals were sacrificed, the eyeballs were fixed, and 16 μm cryostat sections were prepared for microscopic observation. This procedure was performed without the washing step to preserve and detect the circulating NPs regardless of accumulation in specific tissue structures. As shown in Figure 11, NPs inoculated via subconjunctival injection diffused and freely circulated through the choroid and the RPE, and reached the ONL.

**Figure 12:**
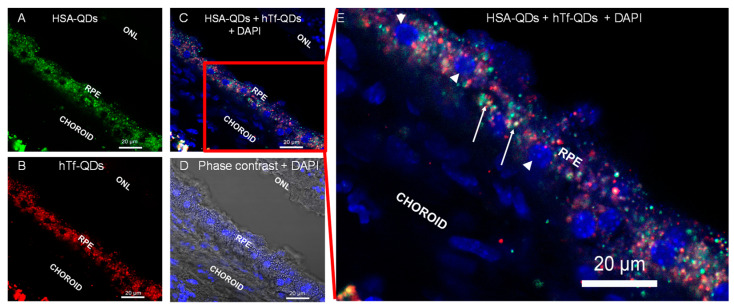
In vivo cellular uptake by RPE cells of HSA-QDs (green filter, Panel (**A**)) and hTf-QDs (red filter, Panel (**B**)) inoculated via subconjunctival injection. Panel (**C**) shows the merge image of HSA-QDs, hTf-QDs, and DAPI-stained nuclei (blue filter). Panel (**D**) shows the merge image of DAPI + Phase contrast. Panel (**E**) is a magnification of the inset shown in (**C**). White arrowheads show DAPI-stained nuclei. White arrows show intracellular co-localization of HSA-QDs and hTf-QDs (yellow dots).

To determine the cellular uptake of circulating NPs, a similar experiment was conducted, and the tissue samples were prepared after a washing step by perfusion with PBS to remove circulating (extracellular) NPs. As shown in Figure 12, HSA-QDs and hTf-QDs were taken only by cells of the RPE.

## 4. Discussion

The corona effect is a critical barrier to the development of effective target recognition and drug delivery via NPs [32]. Very recently it has been proved that multilayer albumin coating of gold NPs, also called biohybrid NPs, avoids the protein corona formation once they are in contact with plasma. This relevant property allows enhanced cell recognition when the NPs are decorated with a molecule able to recognize a specific cellular receptor [21]. The high content of alpha-helix secondary structure of albumin allows high flexibility to perform the coating process of ‘hard’ materials such as inorganic NPs. In addition, it is the main protein component of human plasma and is also responsible for carrying fatty acids onto its several hydrophobic pockets that can also be used for the delivery of exogenous drugs [39]. It was considered that ZnS-coated QDs preserve fluorescence efficiency by stabilizing the crystal; in addition, its ZnS shell interacts with albumin [40,41].

In this work, a simple procedure (Figure 1) was developed for the preparation of a multilayer albumin-coated CdSe/ZnS QDs (first step) with a superficial monolayer shell (second step) of native proteins (e.g., hTf, HSA) that confers specific target recognition without corona effect in serum-containing media (Figure 3). We chose a two-phase system based on the Folch partition to separate proteins, lipids, and nucleotides [42]. In this partition system, the proteins are found at the interface, showing their hydrophobic regions towards the non-polar phase. As a stripping solvent, methanol was chosen due to its intermediate polarity.

Chemical stability is a very important issue for decorated NPs. In this report, a chemical crosslinking was selected. Different cross-linking agents could be used, such as visible light [43] or ionizing radiation [20]. Although the chemistry of glutaraldehyde in aqueous solutions is not fully understood due to the variety of polymers it can generate, this crosslinking agent is one of the most used [44]. The advantages of glutaraldehyde are the reaction conditions in an aqueous environment, near physiological conditions, and gentle reactions with two primary amines. In addition to its availability and low cost, albumin has around 30 exposed primary amines, and therefore it can be easily intermolecular-cross-linked with glutaraldehyde. Considering the potential application to humans, HSA was selected to avoid immunological response and differences in cellular albumin-receptor recognition between species [45,46].

In the second step, the gluta-QDs, which are multilayer albumin-coated NPs activated by glutaraldehyde, were decorated with hTf or HSA to provide cell selectivity and internalization. These decorated NPs are of small size (in the range of 20–90 nm) and preserve the fluorescence properties of CdSe/ZnS QDs (Figure 2, Figure 3 and Figure 4). The differences in size measured between the HSA-QDs and the hTf-QDs are consistent with the size and structure of both proteins. While hTf has approximately 79 kDa and a cylindrical molecular structure [47], the HSA is 66 kDa globular protein with three homologous domains forming a heart-shaped structure [48,49].

The preservation of protein conformation and function of the external monolayer shell was confirmed by (a) heat denaturation of HSA that showed altered electrophoretic mobility (Figure 3A) and (b) by detection of enzymatic activity of AP (Figure 3C). It should be mentioned that successful peptide or protein decoration depends on the interaction between HSA, pH, and ion strength, which must be tuned to each particular case.

The specific cellular uptake was analyzed by taking advantage of the differential expression of HSA and hTf receptors in cell lines. Our experimental data showed that cells expressing low levels of these receptors do not internalize HSA or hTf-QDs. For instance, the SH-SY5Y cell line differentiated into dopaminergic neurons (lower expression of HSA and hTf receptors) did not internalize HSA-QDs or hTf-QDs after 3 d incubation (Figure 5A,B). In contrast, neuroblastoma cells (undifferentiated SH-SY5Y cells, with higher expression of HSA and hTf receptors) internalized these decorated NPs to a great extent (Figure 5C,D). In addition, a higher proportion of hTf-QDs has been taken up compared to HSA-QDs. These results agree with the presence of HSA and hTf receptors in the cell membrane of A549 and undifferentiated SH-SY5Y cells [35,50].

We also compared cellular uptake using two lung cell lines: A549 and 16HB14o- cell cultures, a tumoral and a non-neoplastic cell line, respectively. The bronchial cell line 16HB14o- accumulates lower quantities of hTf-QDs [51], compared to the A549 alveolar cell line that preserves enriched receptors and nanoparticle accumulation [35,52]. The results showed that the NPs enter preferentially in the A549 cell, and at negligible quantities in 16HB14o- cells (Figure 8).

The uptake of decorated NPs was temperature-dependent, indicating an energy-driven process, possibly by an endocytic mechanism. Co-incubation experiment assays in A549 cells showed that HSA-QDs (green) and hTf-QDs (red) only colocalized when they were close to the nucleus. Such co-localization could be associated with late lysosomes (Figure 8). The experimental results showed that the uptake of hTf-QDs is eight-fold higher than the uptake of HSA-QDs. Therefore, we concluded that both NPs do not share the same pathway. According to the literature, the uptake of HSA is caveolin-dependent [53]. Meanwhile, the recycling of the hTf receptor (hTfR) is clathrin-dependent through vesicles that are mobilized by the dynamin mechanism [54]. Many tumors show overexpression of the hTfR in their cells [55], which creates a potential target for drug delivery. The transferrin (hTf) molecule is a 79-kDa glycoprotein that binds to its hTfR at extracellular pH with an affinity from 1 to 100 nM [56]. Its active internalization by tumor cells makes it a good choice for NP targeting for delivering drugs to tumor cells [57]. Finally, the drug-carrier property of decorated NPs was demonstrated by loading hTf-QDs with digitoxin (hTf-QDs-Dix). For drug delivery assays, A549 and 16HBE14o- cells were incubated with hTf-QDs-Dix for 48 h. As shown in Figure 9, (a) hTf-QDs-Dix decreased A549 cell number (>50%) without affecting 16HBE14o- cells demonstrating that multilayer HSA coated NPs retains its ability to bind digitoxin and that hTf decoration delivers enough NPs to A549 cells and (b) the cytotoxicity of Dix-free hTf-QDs was low in both cell lines despite the differential uptake. While the uptake was much higher in A549 cells compared to 16HBE14o- cells (Figure 9C,D), the cytotoxicity was similar in both cell lines (Figure 9D, middle columns). It is relevant to mention that NPs with a cadmium and selenium core displayed significant toxicity in vitro and in vivo likely by releasing elemental Cadmium from the core surface by Oxygen and Hydrogen ions reaching supra micromolar concentration [58]. Oxygen- and hydrogen-triggered cadmium release can be prevented by the shell, resulting in lower toxicity. We speculate that the cytotoxicity observed in our study was due to the release of cadmium into the culture media from QDs with defective shells.

The in vivo circulation and uptake of decorated NPs was demonstrated by studying the mouse retina and its corresponding ARPE-19 cell line. In the mouse retina NPs can be administered through different pathways, offering a versatile model system to test the biodistribution and targeting of HSA-NPs [59,60]. We chose the subconjunctival injection because it is less invasive than the intravitreal injection, allows larger delivery doses, and has been used in clinical settings to deliver drugs to the anterior segment [61]. Our data demonstrated that HSA-QDs and hTf-QDs can be administered via subconjunctival injection, reaching the choroid, and taken up by the RPE cells in agreement with previous results. Pre-clinical experiments have demonstrated the ingestion of NPs by the RPE cells, likely due to their phagocytic capacity [62,63,64]. Bourges et al. showed that the NPs were retained by RPE cells for up to four months after an intravitreal injection [63]. In addition, we demonstrated that both HSA-QDs and hTf-QDs can be administered simultaneously which opens the possibility of co-delivery of different cargos to the same target. The decorated NPs reported in this study, due to their small size (20–90 nm), may be superior to large NPs in terms of diffusion, cellular uptake, and route of administration. For instance, larger particles (200 nm) are retained for long periods in subconjunctival space in comparison to smaller particles (20 nm) [65]. It is relevant to mention that when HSA-QDs and hTf-QDs were administered simultaneously, they did not show extensive co-localization suggesting that the entry may be through different internalization pathways dictated by the protein decorating the NPs.

## 5. Conclusions

In conclusion, using the described simple two-step procedure, we prepared biohybrid decorated NPs with a stable multilayer of albumin with an external protein monolayer shell that retains its biological properties (e.g., binding to its receptor and enzymatic activity). The fluorescent properties of CdSe/ZnS QDs in the core allowed the quantification and intracellular tracking by fluorescence microscopy. Furthermore, these decorated NPs delivered cargo and reached their target cell in vitro and in vivo. We envision this novel biohybrid NP system as a versatile platform to develop target-specific drug delivery to target cells.

## Data Availability

Not applicable.

## Supplementary Materials

The following supporting information can be downloaded at: https://www.mdpi.com/article/10.3390/pharmaceutics15061651/s1. Video S1: Brownian movement of fluorescent nanoparticles video obtained in an invert mic4roscopy at 1.600× magnification.
